# A Dutch perspective on the ESC/EACTS guidelines on myocardial revascularisation

**DOI:** 10.1007/s12471-015-0685-6

**Published:** 2015-04-08

**Authors:** B.E. Claessen, R. van der Schaaf, T. ten Cate, J.J. Piek

**Affiliations:** 1 Department of Cardiology, AMC Heart Center, Academic Medical Center – University of Amsterdam, Amsterdam, The Netherlands; 2 Onze Lieve Vrouwe Gasthuis, Amsterdam, The Netherlands; 3 Radboud University Medical Center, Nijmegen, The Netherlands

To the editor,

## Introduction: the European guidelines from a Dutch perspective

The recently updated 2014 European Society of Cardiology (ESC)/European Association for Cardio-Thoracic Surgery (EACTS) guidelines on myocardial revascularisation provide a framework for decision-making in daily clinical practice [1]. This work requiring an enormous amount of deliberation and discussion by the guideline committee is greatly appreciated. Nonetheless, there are a few recommendations in the current guideline that are not ideally suited to the specific context of myocardial revascularisation in the Netherlands, where decision-making using a heart team consisting of a cardiologist and a cardiothoracic surgeon is deep-rooted [2]. In this document, an addendum to the guidelines is proposed to consolidate these differences between clinical practice in the Netherlands and the broader European context.

## Indications for revascularisation, PCI vs CABG: a case for physiology-guided PCI with next-generation DES

The guidelines state a class III level of evidence (LoE) B recommendation against percutaneous coronary intervention (PCI) in patients with left main disease with a high SYNTAX score (> 32) and three-vessel disease with intermediate (23–32) and high SYNTAX scores. There appears to be insufficient evidence to support this recommendation, given the fact that this is based on only one trial (SYNTAX) in which first-generation drug-eluting stents (DES) were used, which are currently no longer used as they have been superseded by next-generation DES that proved to be superior in terms of repeat revascularisation, stent thrombosis, myocardial infarction and even death [3, 4].

The fact that the guideline advocates assigning PCI or coronary artery bypass grafting (CABG) based upon the purely anatomical SYNTAX score foregoes other important clinical and functional factors that are considered by the typical heart team in the Netherlands. The importance of functional lesion assessment using coronary pressure or flow is not taken into account by the current recommendation. The FAME trials clearly demonstrated the usefulness of fractional flow reserve (FFR)-guided revascularisation in patients with multi-vessel disease [5]. An opportunity to deliver the important message that interrogation, with FFR, of all PCI targets in multi-vessel disease results in better patient outcome is therefore missed. Interrogation of all target stenoses with FFR is also important because it allows calculation of the functional SYNTAX score, which has been shown to be superior to the standard SYNTAX score in terms of risk stratification of patients with multi-vessel coronary artery disease undergoing PCI [6]. Specifically, by only counting lesions that are FFR-positive towards the total of the SYNTAX score, patients can often be assigned to a lower-risk category. Currently ongoing studies such as FAME 3, SYNTAX II, IFR-SWEDEHEART and DEFINE-FLAIR are designed to further refine the use of physiological-guided revascularisation based on FFR or instantaneous wave-free ratio (iFR) in the treatment of multi-vessel coronary artery disease.

Therefore, the final decision on PCI or CABG in complex multi-vessel disease should be made by the heart team whilst taking into account state-of-the art techniques and devices, functional lesion assessment and the specific preferences of individual patients.

## Early invasive strategy in non-ST-elevation ACS

The guidelines recommend an early invasive strategy in patients with non-STE acute coronary syndromes (ACS) in all patients with at least one primary high-risk criterion (GRACE score > 140, relevant rise or fall in troponin or dynamic ST-segment or T-wave changes). This class I recommendation is supported with an LoE A based upon one individual randomised trial and one meta-analysis [7, 8]. However, the TIMACS trial only showed a reduction in a composite endpoint of death, myocardial infarction or stroke in patients with a high GRACE score, which was a finding from a negative trial and should therefore be interpreted with caution. Although one meta-analysis reported a reduction in secondary endpoints (recurrent ischaemia, length of hospital stay), there was no reduction in hard clinical endpoints [8]. This was confirmed in another meta-analysis [9]. Therefore, the early invasive strategy seems to hold no clear benefit over the invasive strategy within 72 hours.

## Recommendations for daily clinical practice in the Netherlands

The recently updated guideline on myocardial revascularisation presents a framework for daily clinical practice, but parts of this guideline cannot be directly applied to daily clinical practice in the Netherlands. We propose the following alternative recommendations:

*Recommendation for the type of revascularisation (CABG or PCI) in patients with stable coronary artery disease with suitable coronary anatomy for both procedures and low predicted surgical mortality:*

Regardless of the extent of coronary artery disease, the decision should be made by the heart team based on the patient’s clinical characteristics, procedural risk (e.g. calculated by Euroscore II or similar risk score models), severity and distribution of coronary artery disease and preferably invasive physiological assessment of all lesions.

*Recommendation for invasive evaluation and revascularisation in NSTE-ACS:*

An invasive strategy is recommended (<72 h after initial presentation) in patients with at least one high-risk criterion or recurrent symptoms. High-risk criteria are: relevant rise or fall in troponin, dynamic ST-segment or T-wave changes, intermediate to high GRACE score, diabetes mellitus, renal insufficiency (eGFR <60 ml/min/1.73 m^2^), left ventricular ejection fraction <40 %, early post-infarction angina, recent PCI and prior CABG. There is currently insufficient evidence to support an early invasive strategy (<24 h), with an exception for patients with an indication for urgent coronary angiography (refractory angina, heart failure, cardiogenic shock, life-threatening ventricular arrhythmias or haemodynamic instability).
